# EEG-Based Brain-Computer Interfaces Using Motor-Imagery: Techniques and Challenges

**DOI:** 10.3390/s19061423

**Published:** 2019-03-22

**Authors:** Natasha Padfield, Jaime Zabalza, Huimin Zhao, Valentin Masero, Jinchang Ren

**Affiliations:** 1 Centre for Signal and Image Processing, University of Strathclyde, Glasgow G1 1XW, UK; natasha.padfield@strath.ac.uk (N.P.); j.zabalza@strath.ac.uk (J.Z.); 2 School of Computer Sciences, Guangdong Polytechnic Normal University, Guangzhou 510665, China; 3 The Guangzhou Key Laboratory of Digital Content Processing and Security Technologies, Guangzhou 510665, China; 4 Department of Computer Systems and Telematics Engineering, Universidad de Extremadura, 06007 Badajoz, Spain; vmasero@unex.es; 5 School of Electrical and Power Engineering, Taiyuan University of Technology, Taiyuan 030024, China

**Keywords:** brain-computer interface (BCI), electroencephalography (EEG), motor-imagery (MI)

## Abstract

Electroencephalography (EEG)-based brain-computer interfaces (BCIs), particularly those using motor-imagery (MI) data, have the potential to become groundbreaking technologies in both clinical and entertainment settings. MI data is generated when a subject imagines the movement of a limb. This paper reviews state-of-the-art signal processing techniques for MI EEG-based BCIs, with a particular focus on the feature extraction, feature selection and classification techniques used. It also summarizes the main applications of EEG-based BCIs, particularly those based on MI data, and finally presents a detailed discussion of the most prevalent challenges impeding the development and commercialization of EEG-based BCIs.

## 1. Introduction

Since the inception of personal computing in the 1970s, engineers have continuously tried to narrow the communication gap between humans and computer technology. This process began with the development of graphical user interfaces (GUIs), computers and mice [1], and has led to ever more intuitive technologies, particularly with the emergence of computational intelligence. Today, the ultimate frontier between humans and computers is being bridged through the use of brain-computer interfaces (BCIs), which enable computers to be intentionally controlled via the monitoring of brain signal activity.

Electroencephalography (EEG) equipment is widely used to record brain signals in BCI systems because it is non-invasive, has high time resolution, potential for mobility in the user and a relatively low cost [2]. Although a BCI can be designed to use EEG signals in a wide variety of ways for control, motor imagery (MI) BCIs, in which users imagine movements occurring in their limbs in order to control the system, have been subject to extensive research [3,4,5,6,7]. This interest is due to their wide potential for applicability in fields such as neurorehabilitation, neuroprosthetics and gaming, where the decoding of users’ thoughts of an imagined movement would be invaluable [2,8].

The aim of this paper is to review a wide selection of signal processing techniques used in MI-based EEG systems, with a particular focus on the state-of-the art regarding feature extraction and feature selection in such systems. It also discusses some of the challenges and limitations encountered during the design and implementation of related signal processing techniques. Furthermore, the paper also summarizes the main applications of EEG-based BCIs and challenges currently faced in the development and commercialization of such BCI systems.

The rest of this paper is organized as follows. Section 2 provides an overview of the fundamental concepts underlying EEG-based BCIs. Section 3 then introduces the main features of MI EEG-based BCIs, and Section 4 goes on to discuss in detail different feature extraction, feature selection and classification techniques utilized in the literature. A case study is presented in Section 5 for evaluating different time-/frequency-domain approaches in controlling motor movement. Section 6 summarizes the different applications for EEG-based BCIs, particularly those based on MI EEG. Finally, Section 7 discusses the main challenges hindering the development and commercialization of EEG-based BCIs.

## 2. Overview of EEG-Based BCIs

This section introduces some of the core aspects of EEG-based BCIs. It explains why EEG is a popular technology for BCIs, along with the generic challenges associated with EEG signal processing. It then introduces the two main classes of EEG-based BCIs, highlighting factors that should be considered when choosing between the two approaches and the challenges inherent to each. Finally, the basic signal characteristics of MI EEG data are summarized, with a brief discussion of the signal processing problems this data can present.

EEG signals are typically used for BCIs due to their high time resolution and the relative ease and cost-effectiveness of brain signal acquisition when compared to other methods such as functional magnetic resonance imaging (fMRI) and magnetoencephalography (MEG) [2]. They are also more portable than fMRI or MEG technologies [9]. However, EEG signals pose processing challenges; since they are non-stationary, they can suffer from external noise and are prone to signal artefacts [2,10]. Furthermore, EEG signals can be affected by the posture and mood of a subject [11]. For example, an upright posture tended to improve the focus and EEG quality during recording [12], and high-frequency content was stronger when users were in an upright position, as opposed to lying down [13]. It was also noted in [12] that postural changes could be used to increase attention in subjects who felt tired.

EEG-based BCIs can be classified into two types: evoked and spontaneous [11], though some works also refer to them as exogenous and endogenous, respectively [2]. In evoked systems, external stimulation, such as visual, auditory or sensory stimulation, is required. The stimuli evoke responses in the brain that are then identified by the BCI system in order to determine the will of the user [14]. In spontaneous BCIs, no external stimulation is required, and control actions are taken based on activity produced as a result of mental activity [2,11]. Table 1 contains examples of typical EEG systems, with details on the application, functionality, and number of subjects involved in testing, where the mean accuracy and information transfer rate were also provided.

The decision between using an evoked or spontaneous system is not always clear, and may require the consideration of the strengths and weaknesses of each approach. Specifically, while evoked systems typically have a higher throughput, require less training and sensors, and can be mastered by a larger number of users when compared to spontaneous systems, they require the user’s gaze to be fixed on the stimuli and this constant concentration can be exhausting [2,9,10].

Typical evoked EEG systems can be separated into two main categories: those dependent on visually evoked potentials (VEPs), brain signals generated in response to a visual stimulus, and event-related potentials (ERPs), brain signals generated in response to sensory or cognitive events [9,14]. Steady-state visually evoked potentials (SSVEPs) are one of the most widely researched areas of VEP-based BCIs because they enable relatively accurate and rapid command input [14] whilst requiring little user training [9]. In such a system, the different options available to a user are displayed as stimuli flickering at unique frequencies, and the user selects an option by focusing on the associated stimulus. The performance of such systems is dependent on the number of stimuli [19], the modulation schemes used, and the hardware used for the stimuli [2,19]. Stawicki et al. [10] conducted a survey of 32 subjects on the usability of an SSVEP-based system, where 66% thought that the system required a lot of concentration to use, 52% thought that the stimuli were annoying, and only 48% considered that the system was easy to use.

When it comes to ERP systems, those based on P300 waves are landmark technologies [2,9]. P300 waves are distinct EEG events related to the categorization or assessment of an external stimulus [20]. However, in such systems, the results need be integrated over several stimuli, which adds to the computational time taken to make decisions, and restricts the maximum throughput of the system [2]. Possible solutions would be to increase the signal-to-noise-ratio (SNR) [21] or find an optimum number of stimuli [22].

A common example of a spontaneous BCI is a MI BCI, which requires the user to imagine the movement of a limb. Such BCIs monitor sensorimotor rhythms (SMRs), which are oscillatory events in EEG signals originating from brain areas associated with preparation, control and carrying out of voluntary motion [9,23]. Brain activity recorded via EEG is typically classified into five different types, depending on the predominant frequency content, *f*, of the signal, summarized as follows: (i) delta activity: *f* < 4 Hz; (ii) theta activity: 4 Hz < *f* < 7 Hz; (iii) alpha activity: 8 Hz < *f* < 12 Hz; (iv) beta activity: 12 < *f* < 30 Hz; and (v) gamma activity: *f* > 30 Hz. In the literature, alpha activity recorded from the sensorimotor region is known as mu activity. Changes in mu and beta activity within EEG signals are used to identify the type of motor imagery task being carried out [9,23]. Gamma activity is reliably used in MI BCIs which use internal electrodes, since gamma signals do not reach the scalp with high enough integrity to be used for MI task identification when recorded using scalp EEG. When activity in a particular band increases, this is called event-related synchronization (ERS), while a decrease in a particular band is called event-related desynchronization (ERD) [23]. ERSs and ERDs can be triggered by motor imagery, motor activity and stimulation of the senses [24,25]. Common classes of movements for MI EEG systems include: left hand movement, right hand movement, movement of the feet and movement of the tongue [26,27,28], since these events have been shown to produce significant and discriminative changes in the EEG signals relative to background EEG [23]. Movement of the feet is often classed as a single class, with no distinction between the left and right foot movement because, as Graimann and Pfurtscheller comment [23], it is impossible to distinguish between left and right foot motor imagery, or between the movements of particular fingers because the cortical areas associated with these distinct movements are too small to generate discriminative ERD and ERS signals. However, Hashimoto and Ushiba illustrated that there is potential for beta activity to be used to discriminate between the left and right MI [29].

The performance of SMR-BCIs is heavily dependent on the neurophysiological and psychological state of the user, with the control of SMR activity being found to be challenging for many users [9,23]. Furthermore, there is a general lack of understanding of the relationship between good and poor performance within BCIs in general, and the neuroanatomic state of a user [30] and BCI performance could have a significant impact on SMR-based BCIs due to their heavy reliance on users successfully learning to consciously generate the required signals. More research is required to understand how these neurological factors affect performance of SMR-BCIs, and how they could possibly be exploited to improve performance [30].

Due to the complex nature of EEG signals, and the strong relationship of signal quality to the mental state of the user, recording EEG data for testing and ensuring that datasets are ‘valid’ is a significant challenge. This is particularly true for MI EEG data, which requires significant focus by the user to generate. In An et al. [31], the performance of a classifier for MI data was analyzed, in which participants carried out MI tasks for an interval of four seconds. They found that during the first two seconds of a MI task, the classification accuracy for a given a particular processing system was at its peak, but for the final two seconds, the classification accuracy decreased. They believed that this was possibly due to subjects losing concentration on the task, resulting in poor-quality EEG data and poor classification results. This highlights two issues: firstly, the validity of MI EEG data may be closely linked with the duration of a task, and thus it would be beneficial to test detectors with data from a complete MI task. The segments of that data should be taken from the beginning and end of the task in order to observe how performance varies. Secondly, future research could investigate how the quality of the EEG data changes during an MI EEG task longer than two seconds, and which kinds of signal processing approaches cope best with longer MI tasks. Zich et al. [32] used fMRI in order to validate MI EEG data, and suggest that fMRI can be used in conjunction with EEG technologies to investigate inter-individual differences in MI data generation. This literature review now focuses on EEG-based MI BCIs in greater depth.

## 3. Introduction to MI EEG-Based BCIs

The aim of this section is to explain why MI EEG signals are used in BCIs, to discuss the inherent challenges presented by the nature of MI EEG data and to introduce the structure of a typical signal processing pipeline for MI EEG data. It also discusses generic technical challenges in this field, including the high dimensionality of multichannel EEG data, the choice between averaged and single-trial results and the choice of pre-processing approach.

MI is widespread in BCI systems because it has naturally occurring discriminative properties and also because signal acquisition is not expensive. Furthermore, MI data in particular can be used to complement rehabilitation therapy following a stroke. This notwithstanding, the processing of MI data is challenging, and most processing and classification approaches are complex, with many approaches suffering from poor classification accuracy since EEG signals are unstable [33,34]. Also, many classifiers fail to consider time-series information [33], even though the inclusion of such data increases classification accuracy [34]. Also, the fact that the MI data of stroke patients is significantly altered when compared to healthy subjects creates challenges in the design of BCIs for post-stroke rehabilitation or therapy [5,35].

**Figure 1:**
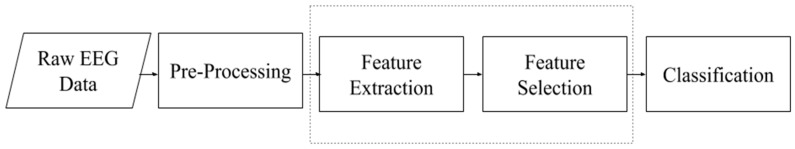
A diagram showing the signal processing carried out in a typical MI EEG-based system.

Figure 1 shows the structure of an EEG-based BCIs for MI applications. In many systems, raw EEG data is pre-processed to remove noise and artefacts [3,11], though not all systems pre-process data [4]. Features are then extracted from the EEG data and the most salient features for classification may be selected. Based on the extracted features, the classifier then identifies which motor movement was imagined by the user. Each section of this diagram will be discussed in greater detail in this paper, with a special focus on feature extraction and selection techniques.

### 3.1. Raw EEG Data

Numerous EEG-based BCIs use data recorded from multiple EEG channels as opposed to a single channel [3]. A key problem when using multichannel data is the high computational costs and possibly poorer performance if feature selection is not used [36]. Future work could involve investigating how data from different channels can be combined or fused using averaging [37], a voting system [38] or PCA [39].

In some areas of BCI research, particularly ERP-based BCIs, salient EEG signal events are often identified in data which has been averaged across subjects or trials [40,41]. Although this approach is widely used in neuroimaging research [41], it has the potential to hide poor performance through the quotation of averaged results [40]. In fact, many studies now use single-trial data, in which results are not averaged across trials [3,42]. These kinds of results are important as they enable the analysis of the variability in performance across trials and can also provide a unique insight into brain activity [40]. Quoting results using single-trial data may also provide a clearer picture of BCI performance in a practical scenario.

### 3.2. Pre-Processing

In the literature, different approaches have been used to reduce the effects of noise in EEG signals with the aim of increasing the accuracy and robustness of BCI systems. Kevric and Subasi [11] argue that linear de-noising approaches, though effective, smooth out sharp transitions in EEG signals, which may result in salient signal characteristics being deteriorated, and they propose that nonlinear filtering techniques such as multiscale principle component analysis (MSPCA) are a better alternative, since they effectively remove noise but preserve sharp transitions [11,43]. MSPCA has been successfully used in a classification system for EEG signals associated with epileptic seizures [44] and another study has successfully merged MSPCA with statistical features for EEG signal processing, with encouraging results [45]. Kevric and Subasi [11] also improved the classification accuracy, in part, for MSPCA compared to when other pre-processing techniques were used.

### 3.3. Feature Extraction, Feature Selection and Classification

The extracted features must capture salient signal characteristics which can be used as a basis for the differentiation between task-specific brain states. Some BCIs involve a process of feature selection, where only the most discriminant of features in a proposed feature set are passed to the classifier with the aim of reducing computation time and increasing accuracy [3,5]. Based on the selected features, the classifier identifies the type of mental task being carried out, and activates the necessary control signals in the BCI system. These control signals could be used, for example, to control the selection of an icon on a graphical user interface, or the movement of a neuroprosthesis. Classification approaches used in the literature include linear discriminant analysis (LDA) [3,4,26,46], support-vector machines (SVMs) [3,4,26,47,48,49,50,51], *k*-nearest neighbor analysis [3,11,51], logistic regression [51], quadratic classifiers [52] and recurrent neural networks (RNNs) [28].

Some systems group together the feature extraction, feature selection and classification tasks within a single signal processing block [53,54,55,56,57,58]. These systems are based on deep learning and largely use a convolutional neural network (CNN) structure [53,54,55,56,58].

The rest of this paper has a particular focus on feature extraction and selection techniques, as well as classification approaches.

### 3.4. Hybrid BCIs Using MI-EEG: New Horizons

A hybrid BCI is one which combines a BCI system with another kind of interface [59], which can either be another BCI [60,61] or some other kind of interface [62]. In the case that the hybrid is a merging of two different BCIs, the two BCIs can both be EEG-based [60], or they can be based on some other technology used to record brain activity [61]. For example, [60] created a paradigm which helped to improve the success of users training in an MI EEG system by using SSVEP as a training aid. Please note that in these kinds of systems, the EEG brain signal responses must generally be largely independent of each other. Conversely, in [61], near-infrared spectroscopy (NIRs) was used in conjunction with EEG signals to identify and classify MI events. Alternatively, an example of a hybrid BCI created by combining a BCI with another kind of interface was reported in [62], where a MI EEG BCI was merged with a sensory interface.

In hybrid BCIs, there are two main ways of combining the signals from the different technologies. The first approach involves considering both signals simultaneously in order to identify the MI task being carried out [59]. For example, in the case of [60], in which the SSVEP-based result and the MI-EEG-based result were both considered at the point of decision-making in the system. In the second approach, the signals from the different interfaces are considered sequentially [59], as in the case of [61], where the NIRs system was used to flag the occurrence of a MI event, and the EEG signals were used to classify the event.

The motivation driving the development of hybrid BCIs is sourced in the desire to create systems with high levels of user literacy, meaning a wide number of users can gain mastery over the system. This is especially important in systems dependent on EEG MI data, since users can struggle to generate the required signals, leading to frustration in training and poor mastery of the system. In fact, combining MI EEG training with SSVEP was found to improve user mastery [60]. The struggles a user may face in generating the required signals may be due to an inherent inability or due to some pathology or condition which inhibits the required brain functioning.

Hybrid BCIs are an emerging field and still face fundamental challenges. Chief among these is choosing the right combinations of signals for a given situation and user [59]. Such a design choice should be made by factoring the abilities and limitations of the user, the environment the system is intended for, portability requirements, the overall cost and the control system, such as a prosthetic, being influenced by the BCI. A second challenge is the decision of how to combine the outputs of the two BCIs, and future work in the area may involve implementing a BCI using the combined and sequential approaches and evaluating them based on speed, information transfer rate, computational cost, usability for the user, accuracy and overall BCI literacy in order to see if there are significant differences in any area, and if so, this information would be used to decide which implementation is best in a given situation.

## 4. Feature Extraction, Feature Selection and Classification in MI EEG-Based BCIs

**Figure 2:**
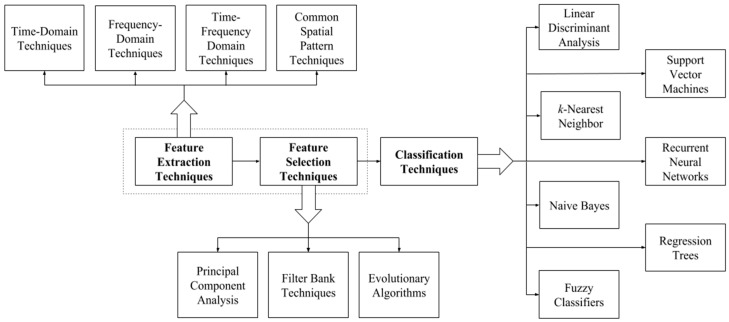
A diagram summarizing some of the feature extraction, feature selection and classification techniques used in MI EEG-based BCIs.

A variety of feature extraction, feature selection and classification techniques are discussed in this section of the paper. The first three subsections discuss the signal processing techniques typically used for feature extraction, feature selection and classification in systems which use distinct signal processing techniques for each task. Figure 2 provides a summary of the most salient techniques discussed in these subsections and Table 2 summarizes different feature extraction, feature selection and classification approaches used in some notable BCI implementations. These works were chosen merely to illustrate a wide variety of the different pipeline structures which have been implemented in the literature. It should be noted that all the works in the Table used the BCI competition III dataset IVa [63], except for the work by Zhou et al. [28], which used the BCI 2003 competition dataset III [64]. The main differences between the datasets was that dataset IVa covers a three-class MI problem with left hand, right hand and right foot movements being used, and dataset III covers a two-class problem involving left or right hand movement. Also, dataset III only contains data from one participant, while dataset IVa has data from five participants. The final subsection discusses the deep learning-based approaches, in which the three signal processing steps are completed within a single processing block.

This whole section deals with the essential challenge of choice of signal processing techniques. To this end, the techniques described in this section are discussed with frequent references to the signal processing challenges and design choice problems which are faced when using the particular techniques for MI EEG processing. Furthermore, the shortcomings and issues associated with particular techniques are also highlighted, as these must be factored when tackling the challenge of pipeline design.

### 4.1. Data and Recording Protocols

Data plays a key role in the training and testing of machine learning systems. It should be noted that the studies discussed in this section of the paper have used different data sets, which all use slightly different recording protocols. The main variations in the datasets are: (i) number of motor imagery tasks considered, with a range between two and four classes possible, (ii) variations in the number of EEG channels recorded and those used in data processing, (iii) variation in the amount of time subjects are allowed to rest between MI tasks, (iv) number of trials and sessions carried out with each subject, (v) number of subjects involved, and (vi) whether an open access or private database was used. These factors should be kept in mind when comparing studies or when applying techniques similar to the literature on new data. Table 2 aimed to show results using various techniques, which were largely generated using the similar data, except for one study.

### 4.2. Feature Extraction

Feature extraction is the signal processing step in which discriminative and non-redundant information is extracted from the EEG data to form a set of features on which classification can be carried out. The most basic feature extraction techniques use time-domain or frequency-domain analysis in order to extract features. Time-frequency analysis is a more advanced and sophisticated feature extraction technique which enables spectral information to be related to the time domain. Finally, analysis in the spatial domain using common spectral patterns is also a prevalent method for feature extraction.

#### 4.2.1. Time-Domain and Frequency-Domain Techniques

As a typical time-domain approach, autoregressive (AR) modelling has been used for feature extraction. In this approach an AR model is fitted to segments of EEG data and the AR coefficients or spectrum are used as features [11,66]. Adaptive autoregressive (AAR) modelling [26,67,68] involves fitting an adaptive model to data segments, and in the literature, model parameters have been estimated using recursive least-squares [69], least mean squares [68] and Kalman filter approaches [70]. Although the Kalman filter is deemed computationally efficient for analyzing EEG signals, its performance is affected by signal artefacts. Other alternative time-domain feature extraction techniques include root-mean-square (RMS) and integrated EEG (IEEG) analysis [71].

Batres-Mendoza et al. [72] proposed a novel approach to time-domain modelling of MI EEG signals based on quaternions. Quaternions, unlike other time-domain techniques used in MI EEG modelling, can represent objects within a three-dimensional space in terms of their orientation and rotation—a property which may be useful when dealing with multichannel EEG data. This technique was found to be effective in extracting features from EEG data for the classification of MI-EEG [72].

Frequency-domain analysis has also been used to extract features from MI EEG data [4,26,73]. While [26] used the fast Fourier transform (FFT) to obtain the power spectrum, [4] used Welch’s method. Welch’s method reduces the noise content in the spectrum when compared to the FFT, but has a lower frequency resolution. Another approach to frequency domain analysis, which did not depend on Fourier theory, was local characteristic-scale decomposition (LCD) [74]. This approach decomposes the signal into intrinsic scale components which have characteristic instantaneous frequencies linked to the characteristics of the original signal.

The spectral analysis for feature extraction is weak as it provides no information relating the frequency content of the signal to the temporal domain. Similarly, time-domain-based analysis ignores spectral features which may be of use for classification.

#### 4.2.2. Time-Frequency Domain Techniques

Time-frequency analysis is powerful since it enables spectral information about an EEG signal to be related to the temporal domain, which is advantageous for BCI technologies since spectral brain activity varies during a period of use of the system as different tasks are carried out [23]. Approaches used for MI EEG analysis include the short-time Fourier transform (STFT) [55], the wavelet transform (WT) [75] and the discrete wavelet transform (DWT) [76]. Decomposition methods such as the WT and the DWT are powerful since different EEG signal frequency bands contain different information about MI actions [11,23], and they can be used to decompose a signal in multiresolution and multiscale [77,78,79]. The DWT and WT are competent in deriving dynamic features, which is particularly important in EEG signals since they are non-stationary, non-linear and non-Gaussian [11]. In [80], the DWT coefficients of the frequency bands of interest were extracted as features, and similarly wavelet packet decomposition (WPD) was used to break-down the EEG signals into low frequency and high frequency components, and the coefficients associated with the frequency bands of interest were then extracted as features. By combining the DWT and AR modelling [76], feature sets are constructed based on wavelet coefficient statistics and 6th order AR coefficients.

Kevric and Subasi [11] conducted a detailed study comparing the performance of decomposition techniques which took higher-order statistics as input features. While first- and second-order statistics have been widely used in biomedical applications [79], they restrict the analysis which can be carried out on nonlinear aspects of the signal. Higher-order statistics enable the representation of signal features when signal behavior diverges from the ideal stationary, linear and Gaussian model, something which lends higher-order statistics an advantage over time-frequency approaches [11,79]. This study is one of very few which gives an in-depth comparison of signal decomposition techniques combined with higher-order statistics for the classification of BCI signals [11]. They compared the performance of three different decomposition methods: empirical mode decomposition, DWT and WPD. These decomposition methods were used to create various sub-band signal components from which 6 features were calculated from the decomposition coefficients, including higher-order statistics in the form of skewness and kurtosis. *k*-nearest neighbor (*k*-NN) analysis was used for classification, with the *k* parameter set to 7. MSPCA was used in pre-processing for noise removal. Kevric and Subasi found that the use of MCSPA and higher-order statistics in feature extraction improved the classification accuracy when compared to approaches that did not use this combination of techniques, and the highest classification accuracy obtained was 92.8%. Furthermore, they found that a higher resolution could be obtained with the WPD coefficients when compared to the DWT coefficients and that the modelling limitations of wavelets were mitigated by using higher-order statistics. Kevric and Subasi also suggest that the technique could have an application in stroke rehabilitation technologies.

Typical classifiers used for classification, including LDA, SVM, *k*-NN and logistic regression do not factor the time-series data present in EEG signals, even though factoring this aspect of the signals can improve classification accuracy, since EEG signals are nonstationary [34]. RNNs are commonly used to exploit the time-series nature of signals, but these networks can suffer from gradient vanishing or gradient explosion during training, and they also have an inherent bias towards new data [28]. To minimize these issues, Zhou et al. [28] used a long-term short-term memory (LSTM) RNN classifier, an approach also used in other studies [81,82,83].

In [28], envelope analysis [84] is also used to extract features from the EEG data. This approach has been used in other works [7,85], as bioelectrical signals naturally exhibit amplitude modulation. Zhou et al. [28] merged the Hilbert transform (HT) and the DWT in order to extract features which are related to the amplitude and frequency modulation present in EEG signals. In the first step of the algorithm, the EEG data is decomposed via the DWT, and afterwards, the wavelet envelope of the decomposed sub-bands was obtained via the HT. The wavelet envelope contained time-series data which was fed into the LSTM classifier. Thus, this method used both envelope information and time-series information, and achieved a high classification accuracy.

#### 4.2.3. Common Spatial Patterns

Common spatial pattern (CSP) is one of the most common feature extraction methods used in MI EEG classification [6,47,50,86]. CSP is a spatial filtering method used to transform EEG data into a new space where the variance of one of the classes is maximized while the variance of the other class is minimized. It is a strong technique for MI EEG processing since different frequency bands of the signal contain different information, and CSP enables the extraction of this information from particular frequency bands. However, pure CSP analysis is not adequate for high-performance MI classification because different subjects exhibit activity in different frequency bands and the optimal frequency band is subject-specific. This means that a wide band of frequencies, typically between 4 Hz and 40 Hz, must be used for MI classification, leading to the inclusion of redundant data being processed [47]. The literature has suggested that optimization of filter band selection could improve the classification accuracy of MI EEG BCIs [48,50,87,88]. However, locating the optimal sub-band using pure CSP is time-consuming [89,90].

There are also various alterations to the CSP method which aim to improve its feature extraction capabilities. The common spatio-spectral pattern approach (CSSP) integrates a finite impulse response (FIR) filter into the CSP algorithm, which was observed to improve performance relative to pure CSP [42]. Common sparse spatio-spectral patterns (CSSSP) [90] is a more refined technique which aims to find spectral patterns which are common across channels, as opposed to the individual spectral patterns in each channel. In sub-band common spatial pattern (SBCSP) [46], EEG is first filtered at different sub-bands, and then CSP features are calculated for each of the bands. LDA is then used to decrease the dimensionality of the sub-bands. This was found to obtain improved classification accuracy when compared to CSP, CSSP and CSSSP [46].

Oikonomou et al. [4] compared the performance of MI classification when using CSP and power spectral density (PSD) for feature extraction, where LDA and SVM were used for classification. The PSD approach was found to outperform the CSP approach for classification of left and right MI tasks. This is possibly because of the high dimensionality feature set obtained via PSD analysis [4]. When using the CSP features, the LDA and SVM classifiers performed similarly. However, SVM was found to significantly outperform LDA when the PSD features were used [4].

### 4.3. Feature Selection

Three main feature selection techniques are discussed in this section: (i) principal component analysis (PCA), (ii) filter bank selection, and (iii) evolutionary algorithms (EAs). Table 3 summarizes the different approaches used, providing information on the mathematical nature of the approaches, average classification accuracy obtained when applying them in the EEG processing pipeline and additional comments about these methods.

#### 4.3.1. Principal Component Analysis (PCA)

Analysis and dimensionality reduction techniques, including PCA [65] and independent component analysis (ICA) [7], have also been applied to MI EEG. PCA has been used for dimensionality reduction and feature selection for improved classification [3]. In some cases [65,75], both PCA and ICA are utilized with other signal processing techniques for feature extraction; for example, in [75], ICA and the WT were used in conjunction with each other in order to extract spatial and time-frequency features.

#### 4.3.2. Filter Bank Selection

This feature selection approach is specific to systems which use CSP and CSSP for feature extraction. Previously, in Section 4.2.3, methods to improve the feature extraction capabilities of traditional CSP analysis were discussed. However, none of the methods discussed thus far exploit the intrinsic link between the frequency bands and CSP features [47]. Filter bank CSP (FBCSP) [48] addresses this by estimating the mutual information contained in the CSP features from the various sub-bands. By choosing those which are most discriminant, selected features are fed into an SVM for classification. Although the system outperformed the SBCSP approach, it utilized multiple sub-bands, leading to a hefty computational cost [47]. To decrease the computational demands of FBCSP, discriminant filter bank CSP (DFBCSP) [88,92] was developed, which considers various overlapping frequency bands, and uses the Fisher ratio to analyze the band power of each sub-band in order to identify the most discriminant sub-bands. This analysis is performed on a single channel of EEG data (C3, C4 or Cz). Sparse filter bank CSP (SFBCSP) [93] also uses multiple frequency bands, but aims to optimize sparse patterns, and features are selected using a supervised technique. In another technique, SBLFB [50], Bayesian learning is employed to select CSP features from multiple EEG sub-bands before feeding them into a SVM classifier. This has resulted in improved performance when compared to state-of-the-art techniques.

Kumar et al. [47] noted that the performance of MI classification depends on the selection of the frequency sub-bands used for feature extraction. They aimed to solve the frequency-band selection problem by building on the DFBCSP approach. Instead of using single-channel data as proposed in the DFBCSP approaches reviewed, data from all available channels was used for the extraction of both CSP and CSSP features from various overlapping sub-bands. Furthermore, Kumar et al. introduced a novel frequency band covering 7-30 Hz. Using the extracted features, mutual information for the bands is then calculated, and the most discriminative filter banks are chosen to be forwarded to the next signal processing stage. In this stage, LDA is used to reduce the dimensionality of the features extracted from each filter bank. Afterwards, the LDA results are joined and fed into an SVM for classification. The performance of this novel technique was compared to that of the CSP, CSSP, FBCSP, DFBSCP, SFBCSP and SBLFB techniques, and was found to have the smallest misclassification rate, and had a strong overall prediction capability. Provided that the model used works exceptionally well, a future improvement to the algorithm could be automatic the learning of the parameters for the filter band.

#### 4.3.3. Evolutionary Algorithms

A key issue in BCI development is the high dimensionality of data during feature extraction. Typical dimensionality reduction and feature selection methods such as PCA and ICA involve complex transformations of features leading to substantial computational demands and a larger sized feature set. These methods often result in low classification accuracy even if the variance of the data is acceptable, possibly because basic feature extraction tends to retain some redundant features. Furthermore, linear transforms tend to be used to decrease the dimensionality of the feature set [3].

Evolutionary algorithms (EA) may offer a possible solution, by enabling features to be selected based on optimization of the classification accuracy of the system. They are promising because in some applications they have been shown to be successful in searching large feature spaces for optimal solutions [3]. EAs such as particle swarm optimization (PSO) [3,49,94], differential evolution (DE) optimization [3,95], artificial bee colony (ABC) optimization [3,96], ant colony optimization (ACO) [3], genetic algorithms (GAs) [49] and the firefly algorithm [74] have been successfully applied for feature selection and reduction.

**Figure 3:**
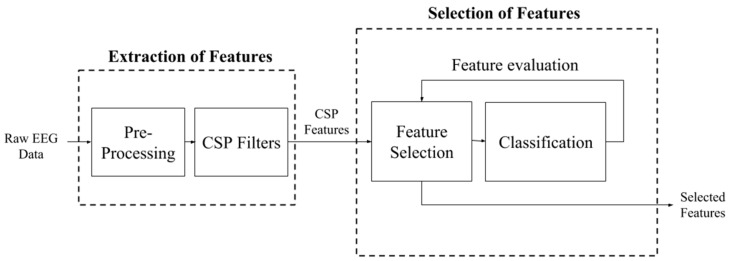
A diagram of the feature extraction and feature selection process proposed in [3].

Baig et al. [3] propose a new feature selection approach based on DE optimization, which aims to decrease computational demands while improving the effectiveness of the feature set by choosing only relevant features. Figure 3 summarizes the flow of the DE-based feature extraction and feature selection process. They implemented a hybrid approach where CSP is used to extract features, a DE algorithm is used to select an optimized subset of features, and only these features are passed onto the classifier. The system was also tested with different computational methods for feature selection, namely PSO, simulated annealing, ACO and ABC optimization. Also, the framework was tested with five different classifiers: LDA, SVM, *k*-NN, naive Bayes and regression trees. Although the suggested algorithm was relatively slow in feature extraction, and the use of wrapper techniques further slowed the system when compared to state-of-the-art approaches, argued that the significant improvement in accuracy far outweighs the slower computations. Furthermore, it should be noted that the EA algorithm is only used to find the optimal feature set for a given application, and thus after selection of the optimal features, the classification problem can be carried out repeatedly using the pre-selected features. Thus, the computational burden of the EA is only suffered once, during the initial feature selection phase, after which the problem becomes one of simple classification.

Zhiping et al. [49] also implemented a PSO-based two-step method for feature selection from MI EEG data. Firstly, the PSO algorithm was used to choose the classifier parameters and relevant features extracted from the EEG data. Afterwards, redundant features were excluded from the selected features via a voting mechanism. This additional voting mechanism was not implemented in any of the other EAs surveyed. In this application, the feature vector was constructed of time-frequency features extracted using the stationary wavelet transform (SWT) and a finite-impulse response (FIR) filter, and an SVM was used for classification. PSO was found to be effective in increasing the speed of the system as well as reducing the number of redundant features and a stable performance. The results obtained using PSO were compared to those obtained using a GA and were found to be superior. The GA approach suffers from a slower learning process than PSO, with PSO taking advantage of gradient information in order to survey trends in order to obtain an appropriate, optimal answer as opposed to GAs, which search within the data for trends [49].

The firefly algorithm, which bears close similarity to PSO, has also been applied to feature selection when using CSP and LCD features [74]. In [74], combining the firefly approach with a learning algorithm was proposed in order to prevent the optimization process getting caught in a local minimum. Although the firefly algorithm has been criticized as being very similar to the PSO algorithm [91], it resulted in higher classification accuracies when compared to similar pipelines using both genetic and adaptive weight PSOs. 

### 4.4. Classification Methods

The aim of this subsection is to provide a brief summary of the various classification techniques used in the literature. SVMs and LDA were observed to be the widely used classifiers in the literature [3,4,26,46,47,48,49,50,51,72], with the performance of the SVM classifier found to be superior when compared to various classifiers such as LDA, *k*-NN, naive Bayes and regression trees [3,4,47]. The average classification accuracy of the proposed method using the SVM classifier was 96.02%, which was a 2% improvement in classification accuracy when compared to state-of-the-art results. LDA was also found to outperform naïve Bayes when CSP and PSD features were used [11]. In [97], it was also found that the SVM classifier with Gaussian kernel outperforms LDA. Baig et al. [3] also found that SVM and LDA were the best classifiers for DE-based feature extraction, with both obtaining a classification accuracy of 95% with a deviation of 0.1.

Both the LDA and SVM approaches may suffer from overfitting; however, these can be mitigated by applying regularization in LDA and through choice of training scheme in the case of SVMs [98] (pp. 9, 336). Although both popular, SVMs and LDA are fundamentally different, with the LDA approach prone to suffer from the curse of dimensionality, something which is absent when using the SVM approach. Although SVM is popularly used in the literature [3,4,26,47,48,49,50,51,72], logistic regression has been found to perform on par with SVM in terms of classification accuracy, obtaining an accuracy of 73.03% compared to 68.97% for SVM. Logistic regression also performed better than *k*-NN and artificial neural network (ANNs) approaches [51]. Although SVM and logistic regression are strong classifiers in MI EEG processing, there is a relationship between the accuracy of the classification, classifier type and the type of features used. In fact, the classification performance of the *k*-NN and ANN approaches can be further improved by using features which are strongly correlated [99].

*k*-NN approaches were also found to be common in the literature [3,11,72]; however, these are memory-based approaches, meaning that a full dataset must be stored in memory and processed all at once. This inevitably increases computational costs when compared to kernel-based methods such as SVM [98] (p. 292). Furthermore, SVMs may be viewed as more powerful than the *k*-NN approach since it constructs an optimization problem which has one global solution which can be calculated in a straightforward way [98] (p. 225).

Quadratic classifiers have not typically been applied to MI EEG processing. However, a quadratic classifier was successfully applied to an EEG classification problem involving the detection of epileptic activity, obtaining an overall classification accuracy of 99% [52]. Future work could investigate the application of quadratic classifiers to MI EEG classification problems.

Computational intelligence methods have also been used for classification. These include deep learning architectures [5,58,100], as well as RNNs [28], which were previously discussed. Lu et al. [101] used a deep neural network constructed using restricted Boltzmann machines and obtained better accuracy than state-of-the-art methods including CSP and FBCSP. Similarly, using a CNN approach, [58] obtained a better classification performance than a FBCSP approach. Future work may involve comparing the performance of the deep learning classifiers in [58,100] with SVM and LDA classifiers. Cheng et al. [5] tried to improve MI classification for data from stroke patients—which deviate from MI data from healthy patients- by using deep neural networks (DNNs) to select the best frequency bands from which to generate features in order to improve classification accuracy. They found that features selected from the identified sub-bands gave better classification accuracies than when selecting features using standard methods. Furthermore, they found that a DNN classifier was often more accurate than an SVM classifier.

Fuzzy classification is another computational intelligence approach used for EEG classification that has gained popularity because EEG classification is a decision-making problem suited for fuzzy logic. These approaches challenge established approaches to EEG signal processing and classification [101], which have already been discussed in this paper. Yang et al. [102] proposed an adaptive neuro-fuzzy interface system (ANFIS) which aimed to classify background EEG recorded from subjects suffering from electrical status epilepticus slow wave sleep (ESES) syndrome and healthy controls using sample entropy and permutation to construct the features. The mean accuracy was reported to be 89%. Alternatively, Herman et al. [103] used an interval type-2 fuzzy logic system, which was designed to accommodate for the non-stationarity inherent to EEG signals. Using 5-fold cross-validation (CV), a classification accuracy of 71.2% was obtained, with the approach outperforming state-of-the-art systems. Finally, Jiang et al. [104] used a Taigi-Sugeno-Kang approach, and applied a multiview learning approach to provide better generalization. Interestingly, Jiang et al. used Friedman rank to evaluate the performance of the detector, which is a metric which was not observed to be widely used in the literature. The multiview learning approach provided better results, giving a Friedman rank of 1, as opposed to the system without multiview learning which obtained a rank of 3.65. Fuzzy classifiers have also been used with CSP features [105].

It is evident that classification techniques based on supervised learning were overwhelmingly favored in the literature when compared to those based on unsupervised learning. Unsupervised techniques have been used mainly for feature selection, as discussed previously in Section 4.2.2. However, unsupervised techniques such as Gaussian mixture models have been used for EEG classification problems outside of MI EEG processing, such as in [37], and could possibly be applied to MI EEG as well in future work.

### 4.5. The Deep Learning Approach

Deep learning can be used to perform the whole pipeline of feature extraction, selection and classification within a single processing block [53,54,55,56,57,58]. The architecture most widely used in MI EEG processing were CNNs [53,54,55,56,58], but RNNs [56], stacked auto encoders (SAEs) [55] and deep belief networks [56] have also been used. Studies have found deep learning to outperform state-of-the-art techniques [53,55,56], including those using CSP features [53] and SVM classification [53,56].

Often, other architectures are combined with the CNN architecture. For example, in [55], a CNN was used for feature extraction while a SAE was used for classification, and in [56], a CNN was used to extract features that were invariant to spectral and spatial changes, whist a LSTM-RNN was used to extract temporal information. Finally, Dai et al. [106] used a CNN to extract features and a variational autoencoder (VAE) for classification, and their implementation was found to outperform the state-of-the art approaches for the databases they tested on.

CNNs hold many advantages for MI EEG data processing [53]: raw data can be input to the system thus removing the need to prior feature extraction and they inherently exploit the hierarchical nature of certain signals and they perform well using large datasets. However, their disadvantages are also evident, since the large number of hyper parameters which must be learnt during training can increase the training time compared to other methods, they can produce incorrect classification results with great certainty [107], and the features learnt can be difficult to understand in the context of the original signal.

It should be noted that CNNs were adopted in EEG signal processing after first being established as a tool in image processing [108]. Thus, when using CNNs for the classification of MI EEG, one of the greatest differences between approaches involves the pre-processing of the input data, which can mainly be divided into two solutions, i.e., either configuring the EEG data as an image [55,56], or not configuring the EEG data as an image [53,54,58].

In approaches which convert the EEG data to an image, a time-frequency domain image is obtained from the data. In [55] this is achieved by segmenting the EEG data with a two-second interval, where each interval corresponds to a particular MI task being performed. The STFT is used to produce a time-frequency image of the task, from which the frequency bands most associated with MI EEG are extracted. The extracted image is then fed into the deep network. In [106] it aimed to preserve the channel relationships between the electrodes used in recording by concatenating STFT time-frequency images generated from each electrode to form a single image. However, the STFT ignores any relationship that can exist between the time-frequency domain and the spatial domain. In [56], it attempts to preserve these possible relationships by considering short time segments and extracting from a given segment the information from three salient frequencies. The data obtained from each band was then projected from the 3D space of the electrodes placed on the scalp to the 2D space of an image, and this projection maintains the spatial relationships between the information from each electrode. The resulting 2D images obtained for each frequency band are then grouped to form an image with three color channels.

Contrastingly, [53] proposes a technique in which raw data is fed into the CNN, and the first layers of the network are devoted to extracting spatial and temporal information. This approach leads to a lower dimensionality input than the image-based approach in [56]. In [53], the CNN was learnt to use spectral characteristics to discriminate between tasks. In [54], a time-consuming pre-processing approach was used, in which the data which has the best markers for MI activity, and the best frequency bands for each subject in the study were selected via visual inspection. Finally, in the pre-processing step proposed by [58], augmented CSP (ACSP) features are extracted from the data. Recall that one of the core issues with CSP feature extraction is the selection of the frequency bands for feature extraction, with many approaches using a wide-band method or a filter-bank method for selection. However, this can result in the loss of important information, and ACSP aims to solve this issue by covering as many frequency bands as possible by varying the partitions between the bands.

Deep learning holds much potential in MI EEG classification. Future work could involve a heavier focus on integrating elements of feature selection. For example, the potential of stacked denoising auto encoders, which has been used to locate robust features [109], could be explored. Also, network structure and training could incorporate feature selection elements such as in [110,111,112], where features which are not strongly discriminative according to some criterion are suppressed by the network. Architectures using statistical tests such as the *t*-test [113] or chi-squared test [114] to identify the most discriminative features could also be investigated. Furthermore, heavier research into architectures aside from CNNs could be carried out, such as into the use of stacked auto encoders for the entire pipeline processing and evolutionary neural networks, which has been shown to hold potential for feature extraction and selection [57].

Section 5 now introduces a case study which provides a practical example of an implementation of an MI EEG data processing pipeline.

## 5. Case Study

A particular case study of EEG data processing is briefly introduced in this section. This case was presented at the *IEEE brain data bank challenge* (BDBC) 2017, hosted in Glasgow [115]. A team representing the University of Strathclyde participated in the challenge achieving the 2nd place award for their work ‘Evaluation of different time and frequency domain approaches for a two-directional imaginary motor movement task’, later extended to [116].

In this competition, participants were allowed to work with any EEG data set, being asked to develop open analysis for novel, creative or informative conclusions. This case study focused on the feature extraction stage, implementing different techniques and comparing their performance in terms of (i) classification accuracy and (ii) computation time required for extracting the features.

In the following subsections, information about the data set used in the experiments along with the proposed data processing and results achieved is provided.

### 5.1. Selected Data Set

For this case study, data set number 4 from the *Brain/neural computer interaction (BNCI) Horizon 2020* site [117] was selected. *BNCI Horizon 2020* is a coordination-and-support action funded within the *European Commission’s Framework Programme 7* [117], with the objective of promoting collaboration and communication among the main players in the BCI field.

**Figure 4:**
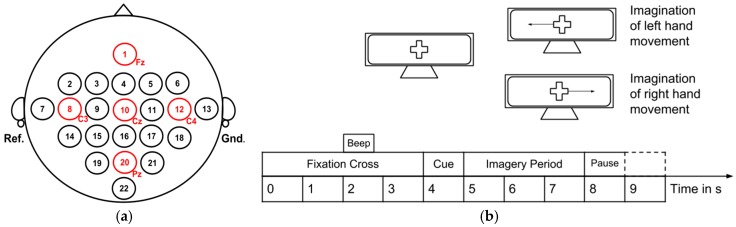
This figure includes information about the acquisition of data set number 4 in *BNCI Horizon 2020* [117], where (**a**) shows the electrodes (C3, C_Z_, C4) placement on the head [118] and (**b**) shows the time scheme paradigm [118] followed during data acquisition.

Data set number 4 was selected for several reasons, including high impact and citation, as well as the data being available in MATLAB files, ready for straightforward use in this platform. The data consists of three bipolar recordings (C3, C_Z_, C4) corresponding to the image shown in Figure 4a. During the acquisition process, subjects under study were told to imagine left hand movement or right hand movement for four seconds after the cue was initialized, roughly one second after hearing a short acoustic tone (beep). Imagination of left or right movement depended on an arrow cue shown in a screen. The time scheme paradigm followed during acquisition is shown in Figure 4b.

Each subject participated in two sessions performed on two different days. Each session contained six runs with ten trials each and two MI classes [118]. A total of 120 trials per session were acquired, leading to 120 repetitions of left MI class and 120 repetitions of right MI class out of the two sessions. This data set also included information related to electrooculography (EOG), but it was not used in the case study. Further details about this data set can be found in [118].

### 5.2. Data Processing Workflow

**Figure 5:**
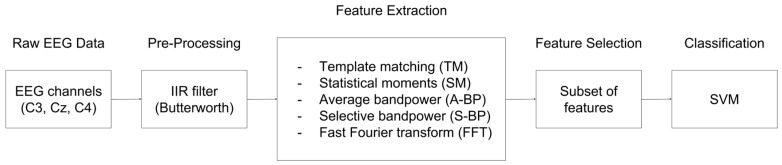
A diagram of the methodology proposed by the University of Strathclyde team in the BDBC 2017 hosted in Glasgow, where different feature extraction techniques were compared under the same conditions.

The main purpose of the data processing was to compare different feature extraction techniques under the same conditions. Figure 5 shows the overall methodology followed for all the techniques, including (i) raw EEG data (C3, C_Z_, C4), (ii) pre-processing based on filtering, (iii) feature extraction (main comparative evaluation), (iv) feature selection, and (v) classification.

The raw EEG evaluated was the same for all the cases, where a common infinite impulse response (IIR) filter (Butterworth) was implemented for pre-processing. Afterwards, different feature extraction techniques were implemented. These were: (i) template matching (TM) [119,120,121] and statistical moments (SM) [122,123,124] in the time-domain, and (ii) average bandpower (A-BP) [25,125,126], selective bandpower (S-BP) [69,118,127] and fast Fourier transform power spectrum (FFT) [128,129,130] in the frequency domain.

TM has been used in previous works related to the detection of salient characteristics in EEG signal processing. For example, [119] used this technique in order to detect transient events within EEG recordings from infants, [120] used it to identify early indications of seizures in epileptic patients and [123] used TM to flap VEP events in a BCI application. SM has also been used in the detection of EEG markers for epileptic seizures [123,124] and as well as for the classification of signals based on modulation [122].

A-BP techniques have been used for the classification of left and right MI [125], as well as for a four-class MI classification problem involving left hand, right hand, feet and tongue MI tasks [25]. They have also been used for the other band-power related EEG classification problems [126]. S-BP has also been used for MI classification tasks [69], as well as classification of other mental tasks including math tasks, geometric rotation, visual counting and letter composition, which are all related to the frequency content of the signal [128]. It has also shown potential to be used in a practical application, in which EEG signals are processed as subjects move around a virtual environment [118].

The FFT is also a powerful frequency-domain analysis tool used in EEG signal processing [128,129,130]. For example, it has been used for the identification of emotional state from EEG data [128,129], as well as for a classification problem involving the identification of Alzheimer’s from EEG data [130].

After the feature extraction stage, a common feature selection was introduced, being based on selecting a particular subset of the extracted features for each case. Finally, the selected features were used to train, validate and test a classification model based on SVM. The classification accuracy of the SVM is directly dependent on the features extracted and, therefore, is different for each of the techniques included in the evaluation.

### 5.3. Performance Comparison

**Figure 6:**
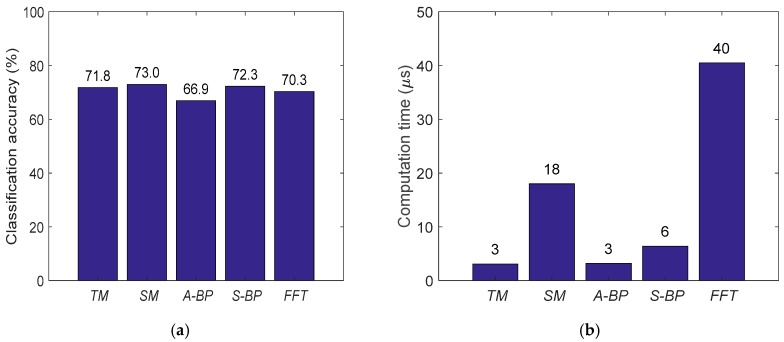
Performance comparison among TM, SM, A-BP, S-BP and FFT feature extraction techniques evaluated under the same conditions, where (**a**) shows the classification accuracy (%) and (**b**) shows the approximated computation time (μs) required to extract the features.

After implementing and running the data processing workflow for each one of the feature extraction techniques mentioned, it was possible to compare the classification accuracy and also the computation time needed in order to extract the features. Figure 6 shows this comparison among TM, SM, A-BP, S-BP and FFT.

Features from all evaluated techniques were able to provide an accuracy close to 70%, where the difference among them is not especially significant. Additionally, there is not much difference among time-domain- and frequency-domain-based techniques, achieving 73% (SM) and 72.3% (S-BP) accuracies, respectively. This fact may highlight the difficulty in processing EEG data, as it seems not feasible to go further than the given level of accuracy, probably due to the inherent EEG data nature, including its acquisition process.

On the other hand, the computation time required for extracting features can make a difference, as some techniques such as TM and A-BP took roughly 3 μs, while others needed up to 40 μs (FFT). Therefore, the main finding from this case study, as presented in the BDBC [115], was to highlight the importance of computational efficiency, where while the accuracy cannot be increased, at least high-speed real-time BCI can be proposed, with potential introduction of GPU and FPGA architectures.

## 6. Applications

Numerous applications exist for BCIs, and the design of a BCI depends on the intended application [5]. Nijholt [8] suggests that there are two main branches of BCI applications: *control* and *monitor*. Control applications are oriented towards manipulating an external device using brain signals while monitor applications are oriented towards identifying the mental and emotional state of the user in order to control the environment they are in or the interface they are using. Practical applications of BCI technologies within and outside the biomedical sphere are discussed in the following subsections, with some mentions of the challenges that can be incurred in the development of such systems.

### 6.1. Biomedical Applications

Roadmaps and research associated with BCI technology has overwhelmingly been focused on medical applications [2,8], with many BCIs intended for the replacement or restoration of central nervous system (CNS) functionality which was lost due to disease or injury [2]. Other BCIs are focused on therapy and motor rehabilitation after illness or trauma to the CNS, in diagnostic applications and finally BCIs are being used in affective computing for biomedical applications. Each of these applications will be discussed in more detail in the following subsections. As well as empowering people suffering from mobility issues or facilitating their recovery, these technologies can also reduce the time and cost of care. A core challenge in the development of such systems is the need to design accurate technologies which can deal with the possibly atypical brain responses which can be the result of illnesses such as stroke [5,35].

#### 6.1.1. Replacement and Restoration of CNS

These technologies can restore or replace functionality of the CNS which was lost due to illnesses such as amyotrophic lateral sclerosis (ALS) and locked-in syndrome, as well as people suffering from paralysis, amputations and loss of CNS functionality due to trauma, such as spinal cord injury. As previously mentioned, the development of such technologies can be challenging due to the altered brain functionality that patients with such conditions can experience.

Currently, many robotic prosthetics depend on myoelectrics, which record electrical signals in muscles. However, such technologies are expensive and assume that nerve connections are largely functional, limiting their applicability for fine control of prosthetics and for patients with CNS injury [131]. BCI-based prosthetics can solve these problems. Müller-Putz and Pfurtschscheller [132] implemented an SSVEP-based robotic arms system with 4 flickering stimuli, each one representing a different function for the arm: later movement to the left or right and opening or closing of the hand. The user selected a movement by looking at the associated stimulus. The system was tested on only 4 subjects, and had a classification accuracy of between 44% and 88%. Such an SSVEP-based system faces the system-specific challenges previously discussed in Section 2.

**Figure 7:**
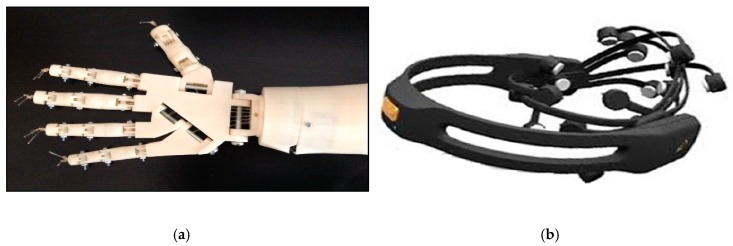
This figure shows the hardware setup used for a low-cost MI-based EEG system e.g., in [133], [136] where (**a**) shows the 3D-printed prosthetic arm which was controlled and (**b**) shows the EEG headset used.

Elstob and Secco [133] propose a low-cost BCI prosthetic arm based on MI, and which has 5 degrees of freedom of movement, as opposed to two. The hardware used in the system is shown in Figure 7; note that an EEG diadem was used. Although this looks significantly different from the standard EEG caps used in clinical research, manufacturers of EEG diadems tend to place electrodes in standard positions according to the 10–20 system. Elstob and Secco reported an accuracy of between 56% and 100%, depending on the movements carried out. MI-based systems may be more suitable than SSVEP systems since they are more intuitive and remove the fatigue associated with looking at the flickering stimuli. However, as previously discussed, MI data can be difficult to generate in the brain.

Müller-Putz et al. [134] also implemented an MI-based robotic arm system, but with 3 degrees of freedom. They also designed a novel 64-electrode sleeve which can be worn by the user which gives feedback via electrical pulses as to the movements carried out, in a process known as functional electrical stimulation (FES). Although the approach is promising, classification accuracies between 37% and 57% were obtained. FES can be used to provide feedback and help to restore aspects of CNS functionality in some patients. A challenge when designing such innovative systems is the harmonious merging of system components, in this case the novel EEG system and the FES aspect of the system, a process that can require numerous incremental system developments. A study [135] into the performance of off-the-shelf anthropomorphic arms controlled via BCI showed that such systems hold promise.

BCI interfaces are also used for wheelchair control. One prototype uses a GUI to list the different movement options available, and P300 signals are processed to identify the intentions of the user. Voznenko et al. [136] used an extended BCI to replace the joystick functionality of a wheelchair. The system enabled the user to choose to control movement using thought, voice or gestures. This was a novel approach in which the three control systems worked in parallel to form an ‘extended’ BCI based on the idea that a user can master a BCI system more effectively when there are multiple control channels. The core challenge in this project was data fusion during decision making when multiple control signals were received at once.

Chella et al. [137] propose a teleoperated robot based on a P300 BCI, enabling remote control of the movement of a robot in a space via a BCI. They suggest one application for this system could be an electronic museum guide, which can send video to the user. Teleoperated systems may suffer from particular challenges when it comes to delays in control signals being sent, particularly when the signals travel over the Internet. In such circumstances, the tele-operated robot, in particular, must have control systems which come into play to prevent it from injuring bystanders or damaging property as it moved through the space.

#### 6.1.2. Therapy, Rehabilitation and Assessment

Robotic BCIs are not only used in neuroprosthetics, but also in therapeutic applications. Stroke rehabilitation can be aided by BCI-controlled robotic arms which guide subjects arm movements [138] and social robots such as that implemented in [139], in which the user imagines motor movements which are then carried out by the robot. A core challenge in the development of rehabilitation technologies is the additional refinement which must be done for systems to pass clinical trials.

BCIs are also being applied to virtual reality (VR) for rehabilitative applications. Luu et al. [140] propose a system which decodes brain activity while subjects walk on a treadmill and provide visual feedback to the user on their movements through a virtual avatar. Such systems may hold promise in post-stroke therapy, and future challenges in the area would involve accurate control of the avatar. Other systems are-based totally in virtual worlds, such as that reported in [141], in which users can control 3D objects within a VR setting via EEG signals. This system is open-source and inexpensive and, with further development, holds promise for application in stroke rehabilitation as well as entertainment. The neurofeedback in such systems would need to be adjusted according to the user and the application, particularly when the neurofeedback from patients with CNS illness or injury is used, as this feedback may not be reliable.

Assessment and diagnosis in a clinical setting can also be complemented by the use of BCIs. In [142] a BCI which users used to play serious games was proposed for the assessment of attention of children with cerebral palsy. Another study [143] investigated EEG features recorded via BCI as an aid for the diagnosis of schizophrenia. Assessment and diagnosis technologies play a critical role in patient wellbeing, and their functionality must be heavily refined to ensure they are safe, appropriate and have industry-standard high-levels of accuracy.

#### 6.1.3. Affective Computing for Biomedical Applications

In affective computing BCIs, users’ mood and psychological state are monitored, possibly in order to manipulate their surrounding environment to enhance or alter that state. For example, Ehrlich et al. [144] implemented closed-loop system in which music is synthesized based on the users’ affective state and then played back to them. Such a system could be used to study human emotions and sensorimotor integration. Affective computing can also be used to help patients with serious neurological disorders to communicate their emotions to the outside world [145].

### 6.2. Non-Biomedical Applications

In recent years, the economic potential of BCI technologies has emerged, particularly in the areas of entertainment, gaming and affective computing [8]. While medical or military applications require researchers to focus on robustness and high efficiency, technologies aimed at entertainment or lifestyle require a heavier focus on enjoyment and social aspects [8]. A key challenge in the design of such systems is how to identify appropriate and interesting systems that would be appealing to commercial users, as well as ensuring they are robust enough for being marketed to a wide and varied audience.

BCIs can also be used to enhance CNS output, and to control the environment the user experiences [146]. Such applications include assistive technologies in the form of affective control of a domestic environment which caters for the emotional needs of the individual in the space as well as in the transport, games and entertainment industries. This close interaction between the BCI and the user, particularly the influence on a user’s mood, can raise ethical issues, since such technologies could be used to exploit user emotions to push targeted marketing or political agendas. Thus, a future challenge would involve outlining regulatory controls on the applications of such technologies, as well as technological safeguards to prevent abuse. BCIs can also be used in order to forward research by driving the development of signal processing algorithms, machine learning, artificial intelligence and hardware interfaces [2,8]. This section investigates some of the prominent non-biomedical applications of BCIs. Less commonly [147], P300 potentials are used to control the BCIs [148,149]. Contrary to many systems depending on SSVEP waves, for systems based on P300 potentials, users do not need to be trained. Also, P300 waves are more robust than SSVEP waves, and can be evoked visually or by audio.

#### 6.2.1. Gaming

BCIs primarily aimed at the gaming industry have become a significant area of research [8,147,150,151]. However, gaming BCIs currently represent a poor replacement for traditional methods of controlling games [147], and this represents a possible area for development. Some current technologies depend on evoked potentials, such as the simple SSVEP-based implementation presented in [152] and the more advanced system in [153], which combined SSVEP data and MI data to control a version of Tetris. EEG data has also been used to control difficulty level in multiplayer games, by triggering dynamic difficulty adjustment (DDA), which increases difficulty for strong players and decreases difficulty for weaker players. In the system, EEG data was used to monitor each players’ personal excitement level and activate DDA when the players experience a drop in excitement, to increase engagement [154]. Refining the algorithms that govern the behavior of the game is a significant problem when building such systems. In recent years, commercial BCI-based systems have been emerging in the gaming market [155].

#### 6.2.2. Industry and Transport

Industrial robotics is another area of application for EEG-based BCIs [155], and such technologies can improve safety in the workplace by keeping humans away from dangerous activities. Such systems could replace tedious button and joystick systems used to train robots in industrial settings, and they can also be used to monitor when a user is too tired or unwell to operate the machinery and take appropriate steps to mitigate any danger, such as by stopping a piece of machinery [155]. Similar BCIs which monitor awareness can also be applied to transport, in order to monitor the fatigue of drivers [156] and monitor to improve the performance of airline pilots [157]. Such systems are used in critical applications, and poor decisions can be expensive in terms of both the human life and monetary burdens on the entities involved. Thus, a key issue in such BCIs is to ensure robustness, reliability and constituent high accuracy despite the fact that EEG data is highly nonlinear and noisy, and prone to inter and intra-individual changeability.

#### 6.2.3. Art

Wadeston et al. [158] identified four different types of BCIs for artistic applications: passive, selective, direct and collaborative. Passive artistic BCIs require no active input from the user and merely select which pre-programmed response to output based on the brain activity of the user. For example, in the BMCI piano [159], the passive brain signals of the user determine which pieces of music are played, as well as the volume and tempo. In selective systems, the user has some restricted control over the system but are still not having a leading role in the creative output. For example, in the drawing application proposed by Todd et al. [160], four SSVEP stimuli are used to enable the user to choose which type of shape is drawn on screen; however, the application decides on where the shape is positioned and its color. Direct artistic BCIs give users much higher levels of control, typically enabling them to select options from detailed menus, with options including things such as brush type and controlling brush stroke movements [161]. Finally, collaborative systems can be controlled by multiple users at once [160]. A future development for artistic BCIs could be a merging with virtual reality, in which designers can meet, collaborate and create designs in a virtual space. The design of artistic BCIs involves understanding the artistic process to ensure that the technology is designed to be a help rather than a hindrance.

## 7. Challenges and Future Directions

Although research into BCI technology has been ongoing for the last 20 years, these technologies have remained largely confined to a research environment and have yet to infiltrate into clinical and home settings. This section discusses the main challenges preventing the widespread adoption of BCIs, which were divided into five categories: (i) challenges faced during the research and development of BCIs, (ii) challenges which impede commercialization, (iii) flaws in the testing approaches commonly seen in the literature, (iv) issues encountered during BCI use which may impede their widespread uptake, and (v) ethical issues.

### 7.1. Challenges Faced in Research and Development

The research and development of BCI interfaces, particularly those based on MI EEG, are fraught with signal processing challenges. These include identifying the most effective techniques for feature extraction and selection, which is challenging due to the highly non-linear, non-stationary and artefact-prone nature of EEG data. Other challenges include data fusion, particularly how the data from different EEG channels can be combined in order to reduce data dimensionality and also possibly improve the classification results. Further investigations are also needed to identify the best classification techniques for the selected features. Research into features and classifiers should also focus on identifying the best methods to be used for patients with CNS injury or illness, as the MI EEG characteristics of such patients can differ from that of healthy individuals.

Research also needs to investigate more effective training approaches. For a BCI to be used by a particular subject, a large number of training trials are typically required from that particular subject, leading to the calibration stage being unacceptably time consuming for a practical system. Thus, studies focused on reducing the calibration time are required. Attempts have been made to decrease the training time by using the covariance matrices associated with CSP features extracted from EEG trials to help the decoding of EEG signals [48,55,58]. However, these approaches fail to exploit the geometry of the covariance matrix, even though this can be used to extract salient information from EEG data [162]. The geometric properties of the covariance matrices exist in the symmetrical positive definite (SPD) space, and Singh et al. [162] developed a framework which used SPD characteristics to reduce calibration time. This framework outperformed other methods previously tested on the IVa dataset. Other avenues of research to reduce the calibration time may involve developing EEG models which are more generalizable. 

Finally, a hallmark challenge in the research and development of BCIs is the design of dependable systems with a stable performance, that can be used by a wide variety of users, with different mental states and in different environments.

### 7.2. Challenges Impeding Commercialization

The commercialization of BCIs is impeded by two main obstacles: the first is concerned with technical barriers which can be solved through the development of more robust, efficient and accurate signal processing infrastructures, and the second involves adapting lab-based technologies for use in the wider world. These two issues are discussed in greater depth in this section.

#### 7.2.1. Technical Barriers to Commercialization

To obtain a clear picture of the barriers preventing the commercialization of BCI interfaces, Vansteensel et al. [163] sent out a questionnaire to over 3500 BCI researchers worldwide, 95% of which worked in BCIs related to EEG or electromyography (EMG) technologies. Overall, the researchers surveyed were confident that BCIs, particularly those to replace or enhance brain functionality, could be commercialized and were realizable within the next 5 to 10 years. The survey indicated that, in the case of non-invasive BCIs, major technological developments were needed in sensors, overall system performance and user-friendliness, while in the case of invasive BCIs, there was a need to develop completely implantable systems, improve system robustness and performance, and clinical trials would need to be carried out to ensure the safety of such systems.

Van Steen and Kristo [2] also suggested research in BCI technology would need to focus on improving bit rates [164], improving signal processing techniques and exploring classification approaches. On the macro-scale, they propose formulating novel approaches to overall BCI system design and the types of control systems used.

#### 7.2.2. Adapting Lab-Based Technologies for the Wider World

One of the biggest leaps in commercialization of BCIs is adapting the interfaces used in the lab for use in the wider world. Although BCIs hold potential to be applied to various areas including home automation [165,166], prosthetics [2,8,133,134], rehabilitation [138,139,140], gaming [8,147,150,151], transport [157,158], education [167], VR [142], artistic computing [159,160,161,162], and possibly even virtual assistants based on affective computing, the leap to creating viable products involves considering several factors. These factors mainly include: (i) choice of technology, (ii) general appeal, (iii) intuitivism, (iv) usability and reliability and (v) cost [2,59,168,169,170,171]. Each of these factors will be discussed in more detail in this section. However, when designers make decisions on any of these factors, it is very important to consider the particular situation and environment in which the technology may be used. For example, the design requirements may be different when designing an affordable technology for the control of a television in a home environment when compared to a BCI system for monitoring the alertness of a pilot in a plane. Similarly, designers must factor in the health of the users; technologies designed for rehabilitation or restoration of lost CNS functionality will often have different or additional requirements when compared to technologies intended for healthy users. Thus, during the earliest stages of system design, it is important that system designers carry out an in-depth analysis of the basic requirements of the system, factoring the environment, situation and target audience.

The choice of technology is the first and arguably the most fundamental step taken when designing a human-computer interface, particularly one intended for use in practical situations. The choice of technology involves first considering all the possible hardware options for the interface, including EEG, NIRs, EMG, EOG and other type of eye-gaze tracking technology, as well as hybrid combinations of these technologies. At this stage, it may be determined that a BCI is not the best option for the commercial application in mind, and an alternative type of technology should be considered. When considering a BCI technology, the choice between an evoked and spontaneous system needs be made, and it is important to factor the trade-offs between these two technologies, which were discussed in Section 2. The following discussions are framed assuming that an EEG-based technology was chosen.

Intuitivism is an essential element for any system to be commercializable in the long-term. Intuitivism is linked to the choice of brain signals used, which should be application-appropriate. For example, MI-based technologies may be highly suited for the control of a prosthetic limb or a remote robotic arm since the concept of bodily movement would be intuitive in this kind of scenario. However, for the control of a digital radio, an SSVEP-based system with a 4-option menu consisting of station-up, station-down, volume-up and volume down options may be more intuitive than a MI-based system with imagined movements being associated with television controls. This sense of intuitivism should be factored during the earliest decision phase, when choices of technology and BCI type are being made.

General appeal is another factor underlying all aspects of the design process, and covers anything that may affect the initial impression of the user in relation to the system. The portability of the system is important: headsets which use Bluetooth or Wi-Fi enable users to move around while still being able to use the system, as compared to a wired headset which restricts movements. The aesthetic appeal of the headset and GUIs used would also affect uptake of the technologies. Furthermore, a choice can be made about the type of electrodes used: whether wet or dry electrodes should be used. For a practical application, dry electrodes would be more appropriate as they do not involve the hassle of placing electro-conductive gel between the scalp electrodes, and there is no residue of gel left in the hair after use. However, there is open debate on whether dry electrodes provide the same quality of signal, with some research suggesting dry electrodes produce signals which are noisier and more prone to artifacts than wet electrodes [172], while other research suggests that the signal quality is similar for both types of electrodes [173]. Recently, water-based electrodes have also been studied. It was observed in [174] that in subjects with shorter hair, water-based and dry electrodes performed comparably to gel-based electrodes, and it was suggested that with further refinement of the electrodes for subjects of varying hair length, water-based and dry electrodes held potential to be used instead of gel electrodes. Until there is a more conclusive consensus on the issue, or the technologies available from different manufacturers become more homogenous in terms of recording capabilities, researchers aiming to develop practical systems using dry electrodes should study the signal processing capabilities of the specific EEG systems and electrodes available to them in order to assess whether the signal quality of appropriate, following constraints suggested in the research [172], and studying in particular the noise and artefacts produced in the EEG signals, as in [172,173].

To be successful on the market, BCI systems must be highly usable and reliable. Usability covers design ergonomics, as well as ease-of-use and the time taken for a new user to train on the system- which should be minimal. The ideal technology would be one which average users can pick up and learn how to use through intuition or following a short tutorial, similar to when buying a new mobile. The systems must also be user-friendly and have in-built safeguards to prevent dangerous use. Furthermore, systems need to also be reliable, with users feeling that the technology is dependable and provides stable results following the initial learning period associated with the new technology. The system should also be reliable when used in the multisensory environments in which it is targeted to be used, such as a noisy family home, a busy design studio or the changeable atmosphere of an emergency operating theatre. These multisensory environments may alter the expected brain signals when compared to controlled lab recordings, and thorough development and testing of such systems would need to be carried out. The state of the user during use may also need to be factored, as heartrate and cortisol levels—a hormone associated with stress—are known to interact with EEG signal response [175,176]. Seo and Lee found a significant positive correlation between increased power in the beta band—which is associated with MI EEG data—and cortisol level [176].

Finally, cost is another significant obstacle. The average budget of the expected end users should be factored early on, as well as possible economies of scale, as this will determine the types of recording equipment and sensors that can be used, as well as the software and any compliance issues related to the target audience. Although the aim should always be to provide the best trade-off between cost and performance, the price ranges affordable by students, families, private healthcare companies, the military, start-ups and large technological corporations, to name but a few possible target audiences, vary just as widely as their needs. Researchers serious on developing new technologies to target a particular audience would do well to consider market research prior to beginning the design process of the system.

The commercialization of BCIs would also require the establishment of industry standards, particularly in terms of databases used for benchmarking, EEG recoding equipment and software applications [177]. International roadmap projects such as *BNCI Horizon 2020* [178] have attempted to improve communication within industry and research, so that such issues can be resolved.

### 7.3. A Flawed Testing Process

To implement these recommended improvements, extensive testing on wide populations is required, but the testing process in itself is often flawed. In the literature, a wide array of performance measures have been used to evaluate BCIs, and the lack of a standard approach or single metric to quantify the general performance of a system means limited comparison can be carried out among the systems in the literature [179]. Furthermore, the reporting of basic statistics such as accuracy may obscure prominent issues in the BCI system such as the trade-off between accuracy and speed, and to remedy this, global performance measures such as the utility metric have been recommended in [179,180].

Flaws in widely adopted testing approaches are not limited to the choice of performance metrics, but also the testing data used. Many BCIs which are developed to replace or restore CNS functionality are tested on healthy subjects within a laboratory environment. However, this can lead to unrealistically positive results if typical end-users of these systems would be patients with functional disabilities which have resulted from damage to CNS tissue, for example patients with spinal cord injuries, as BCI performance tends to be poorer with these subjects when compared to healthy controls. Thus, researchers should factor the needs of users with CNS damage during the design and testing of BCI systems for restoring CNS functionality, as was done in [181]. Although there has been research into the design of rehabilitation technologies based on MI for patients following a stroke [5,182,183,184], and in people with ALS [185], there are a plethora of other illnesses or conditions which can affect brain and CNS functioning. These may impede the use of EEG-based BCIs by users who could benefit significantly from them. Conditions which affect the brain or CNS, but have yet to be investigated in-depth in relation to MI EEG-based BCIs, include mild cognitive impairment, diabetes causing loss of cognition, specific types of brain trauma, impairments due to disk and hernia problems, locked-in syndrome, multiple sclerosis, Huntington’s disease and Parkinson’s disease. A comprehensive database of MI EEG data from such patients, as well as healthy controls, all performing the same tasks, would provide a wealth of data for research so the robustness of technologies can be tested, and new solutions found if they fail users with certain conditions.

Even data from healthy subjects tends to be recorded in controlled lab environments. Although this data has an important role in the initial evaluation of signal processing techniques, the development of more robust and commercializable systems would involve testing under stressful conditions. As previously mentioned, heartrate and cortisol can affect the quality of EEG signals, in environments outside the lab, both indoors and outdoors, with varying sensory stimulation in the environment such as noises, movements and smells, and with the subjects sat in different postures. A database of recording data from the same set of subjects performing the same set of MI tasks in all these different scenarios, as well as in a controlled laboratory setting would be a valuable starting point in order to find out how the effectiveness of MI data processing techniques can vary between different scenarios, and how these techniques can be made more robust. Also, for BCIs to be used in practical applications, more research is needed into how external factors related to the individual’s lifestyle can affect BCI performance; for example, consumption of sugar-based drinks has been found to decrease performance of a BCI [186].

### 7.4. Issues with BCI Use

BCI illiteracy is another impediment to the universal adoption of BCI technologies, particularly in EEG-based interfaces. Illiteracy occurs when a user is unable to control a BCI because they do not manage to produce the high-quality brain signals required [177,187]. EEG signal quality, as well as overall mastery of the BCI, can be improved by using an interactive, co-learning approach which provides the user with feedback, such as audio or visual feedback, as they use the system [177].

Long-term use of BCIs requires repeated use of particular neural pathways, and more research needs to be carried out in order to identify the possible health implications or changes in brain functionality due to this. For example, studies have indicated that long-term use of external actuators through BCIs has led to restructuring of the brain’s map of the body, with the actuators being perceived by the brain as an extension to the subject’s body [177,188].

### 7.5. Ethical Issues

Ethical standards must also be established in order to guide the development of BCI technology into the future. Such ethics would establish liability in the case of accidents occurring during the use of controlled apparatus, as well as deal with the appropriate use of bio-signal data and privacy. The BCI Society aims to meet some of these needs by releasing standards and guidelines associated with ethical issues [8,177,178].

## Figures and Tables

**Table 1:** A table containing examples of evoked and spontaneous BCIs.

Type	Class	Example/Application	Display & Function	No of Subjects	Mean Accuracy	ITR ^1^
Evoked	VEP	SSVEP/Speller [10]	Look at one of 30 flickering target stimuli associated with desired character	32	90.81%	35.78 bpm
	ERP	P300/Speller [15]	Focus on the desired letter until it next flashes	15	69.28%	20.91 bpm
		Auditory/Speller [16]	Spatial auditory cues were used to aid the use of an on-screen speller	21	86.1%	5.26 bpm/0.94 char/min
Spontaneous	N/A	Blinks/Virtual keyboard [17]	Choose from 29 characters using eye blinks to navigate/select	14	N/A	1 char/min
		Motor imagery (MI)/Exoskeleton control [18]	Control an exoskeleton of the upper limbs using right and left hand MI	4	84.29%	N/A

^1^ ITR—information transfer rate.

**Table 2:** A comparison of the different combinations BCI structures used in the literature, including features extracted, feature selection approach if used and classification method.

Paper	Feature Extraction Method ^1^	Feature Selection Method ^2^	Classification Method ^3^	Classification Accuracy ^7^
Rodríguez-Bermúdez & García-Laencina, 2012 [26]	AAR modelling, PSD	LARS/LOO-Press Criterion	LDA with regularization	62.2% (AAR), 69.4% (PSD)
Kevric & Subasi, 2017 [11]	Empirical mode decomposition, DWT, WPD ^4^	Kaiser criterion	*k*-NN	92.8% (WPD) ^6^
Zhou et al., 2018 [28]	Envelope analysis with DWT & Hilbert transform	None	RNN LSTM classifier	91.43%
Kumar et al., 2017 [47]	CSP & CSSP ^5^	None, FBCSP, DFBCSP, SFBCSP, SBLFB, DFBCSP-MI ^4^	SVM	Classification accuracy was not quoted.
Yu et al., 2014 [65]	CSP	PCA	SVM	76.34%
Baig et al., 2017 [3]	CSP	PSO, simulated annealing, ABC optimization, ACO, DE ^4^	LDA, SVM, *k*-NN, naive Bayes, regression trees ^4^	90.4% (PSO), 87.44% (simulated annealing), 94.48% (ABC optimization), 84.54% (ACO), 95% DE ^8^

^1^ Associated acronyms: AAR—adaptive autoregressive, PSD—power spectral density, DWT—discrete wavelet transform, WPD—wavelet packet decomposition, CSP—common spatial pattern, CSSP—common spatio-spectral pattern. ^2^ Associated acronyms: FBCSP—filter bank CSP, DFBCSP—discriminative FBCSP, SFBCSP—selective FBCSP, SBLFB—sparse Bayesian learning FB, DFBCSP—MI—DFBCSP with mutual information, PCA—principal component analysis, PSO—particle swarm optimization, ABC—artificial bee colony, ACO—ant colony optimization, DE—differential evolution. ^3^ Associated acronyms: LDA—linear discriminant analysis, *k*-NN—*k*- nearest neighbor, RNN LSTM—recurrent neural network long-short-term memory, SVM—support vector machine. ^4^ The comma between the terms denotes that the methods listed were tested separately. ^5^ The ‘&’ between the terms denotes that the feature vector was constructed of both types of features. ^6^ Mean accuracy only available for the proposed method, which consisted of the WPD combined with higher-order statistics and multiscale principal component analysis for noise removal. Preliminary tested found WPD to be superior to empirical mode decomposition and DWT. ^7^ Mean classification accuracy except result from Zhou et al., for which best accuracy only was quoted. ^8^ Averaged across the results for individual subjects.

**Table 3:** A summary of the different feature selection techniques discussed in this subsection.

Method	Type	Mean Classification Accuracy ^1^	Comments
Principal component analysis (PCA) [65]	Statistical	76.34%	Assumes components with the highest variance have the most information.
Filter Bank Selection [47]	Various	N/A ^2^	Used only for frequency band selection with CSP [47]
Particle-Swarm Optimization (PSO) [3]	Metaheuristic	90.4%	Strong Directional search and population-based search with exploration and exploitation [91].
Simulated Annealing [3]	Probabilistic	87.44%	Aims to find the global maximum through a random search. [3]
Artificial Bee-Colony (ABC) Optimization [3]	Metaheuristic	94.48%	Searches regions of the solution space in turn in order to find the fittest individual in each region. [91]
Ant Colony Optimization (ACO) [3]	Metaheuristic	84.54%	Uses common concepts of directional and population-based search but introduces search space marking. [91]
Differential Evolution (DE) [3]	Metaheuristic	95%	Similar to GAs, with a strong capability of convergence. [3]
Firefly Algorithm [74]	Metaheuristic	70.2%	Can get stuck in local minima, [74] introduced a learning algorithm to prevent this.
Genetic Algorithm (GA) [74]	Metaheuristic	59.85%	Slower than a PSO approach [49], [49] found that PSO was more accurate.

^1^ The performance of the feature selection method can only be truly compared quantitatively to other methods when they were tested with the same data, feature vector and classifier. Thus, although the classification accuracies are listed, true comparisons can only be made when the references associated with the selection methods in the first column are the same. ^2^ Paper did not quote classification accuracy.
